# Withdrawal of caffeine after its chronic administration modifies the antidepressant-like activity of atypical antidepressants in mice. Changes in cortical expression of *Comt*, *Slc6a15* and *Adora1* genes

**DOI:** 10.1007/s00213-018-4940-6

**Published:** 2018-06-07

**Authors:** Aleksandra Szopa, Ewa Poleszak, Urszula Doboszewska, Mariola Herbet, Katarzyna Świąder, Elżbieta Wyska, Anna Serefko, Aleksandra Wlaź, Agnieszka Korga, Marta Ostrowska, Piotr Juś, Szymon Jedynak, Jarosław Dudka, Piotr Wlaź

**Affiliations:** 1 Chair and Department of Applied Pharmacy, Medical University of Lublin, Chodźki 1, 20-093 Lublin, Poland; 2 Department of Animal Physiology, Institute of Biology and Biochemistry, Faculty of Biology and Biotechnology, Maria Curie-Skłodowska University, Akademicka 19, 20-033 Lublin, Poland; 3 Chair and Department of Toxicology, Medical University of Lublin, Chodźki 8, 20-093 Lublin, Poland; 4 Department of Pharmacokinetics and Physical Pharmacy, Jagiellonian University Medical College, Medyczna 9, 30-688 Kraków, Poland; 5 Department of Pathophysiology, Medical University of Lublin, Jaczewskiego 8, 20-090 Lublin, Poland

**Keywords:** Caffeine, Agomelatine, Mianserin, *Slc6a15*, *Adora1*, *Comt*, Mice

## Abstract

**Rationale:**

Depressed patients often present increased consumption of caffeine.

**Objectives:**

We aimed to investigate the effects of chronic treatment with caffeine (5 mg/kg, twice daily for 14 days) on the activity of single, ineffective doses of agomelatine (20 mg/kg) or mianserin (10 mg/kg) given on day 15 alone or simultaneously with caffeine.

**Methods:**

We used the forced swim test (FST), tail suspension test (TST), and locomotor activity test in mice and quantitative real-time PCR analysis of the selected genes in the cerebral cortex (Cx).

**Results:**

There were no changes in the immobility time between mice that received saline and caffeine for 14 days. Administration of agomelatine or mianserin on day 15 did not produce an antidepressant-like effect, but such effect was observed after administration of agomelatine or mianserin simultaneously with caffeine on day 15, in both mice that received saline and caffeine for 14 days. In mice treated with caffeine for 14 days, joint administration of agomelatine or mianserin and caffeine on day 15 decreased solute carrier family 6, member 15 (*Slc6a15*), messenger RNA (mRNA) level in the Cx, compared to the group which received only the respective antidepressant on this day. Moreover, in mice treated with caffeine for 14 days, joint administration of mianserin and caffeine on day 15 decreased adenosine A1 receptor (*Adora1*) and catechol-*O*-methyltransferase (*Comt*) mRNA level in the Cx, compared to the group which received mianserin without caffeine on this day.

**Conclusions:**

Withdrawal of caffeine after its chronic intake can modify the activity of antidepressants. *Adora1*, *Slc6a15*, and *Comt* may be involved in the antidepressant-like effect observed after joint administration of caffeine and mianserin or agomelatine, following chronic treatment with caffeine.

## Introduction

Caffeine (1,3,7-trimethylxanthine) is the world’s most frequently used psychoactive substance. It is derived mainly from the dietary sources, i.e., coffee and tea, while its other sources include soft drinks like cola and energy drinks as well as cold medications, analgesics, and dietary supplements. It is widely accepted that caffeine produces a biphasic effect. Low to moderate doses (50–300 mg) induce behavioral stimulation: physical endurance, reduction of fatigue, and enhancement of mental alertness and concentration, while higher doses cause aversion, irritability, and discomfort (Benowitz 1990; Heckman et al. 2010). In terms of the underlying mechanisms, a low dose of caffeine acts as a nonselective antagonist of adenosine acting at A_1_ and A_2A_ adenosine receptors, while adenosine receptors are not required for the effects observed after administration of its higher doses (Fredholm et al. 2016). It should be noted that adenosine via inhibitory A_1_ and excitatory A_2A_ receptors regulates the release of all known neurotransmitters (Sebastião and Ribeiro 2009), which points to the role of caffeine at low, usually consumed doses in diseases associated with neurotransmitters imbalance, e.g., depression and, presumably, its treatment.

The prevalence of depression is increasing, while its available treatment options do not provide satisfactory effects in a large percent of patients (Trivedi et al. 2006). Therefore, augmentation strategies including modifiable lifestyle behaviors, along with searching for new pharmacological treatment possibilities, are required. Of note, sleep disturbances (insomnia or hypersomnia), psychomotor retardation, fatigue or loss of energy, and diminished ability to think or concentrate are among criteria for a major depressive episode (American Psychiatric Association 2013). Depressed patients often present early morning wakening and daytime fatigue, which may lead to increased consumption of caffeine. Furthermore, they are often prescribed either antidepressants with sleep-promoting properties or sleep medications in addition to antidepressant drugs, which may have a secondary effect on sleep, related to oversedation (Germain and Kupfer 2008; Wichniak et al. 2017) and may also result in increased intake of caffeine.

Considering the above, our study was undertaken with a view to elucidating the effects of chronic exposure to low doses of caffeine and its withdrawal on the activity of single, ineffective doses of antidepressants: agomelatine, which restores sleep-wake pattern and resynchronizes the circadian system (Guardiola-Lemaitre et al. 2014), and mianserin, which possesses sedative properties (Ramaekers et al. 1992). Antidepressants, through changes at the molecular level, can modulate the activity of neurotransmitter systems. Studies show that these drugs, by affecting the activity of neurotransmitters, can also lead to activation or deactivation of certain genes (Tardito et al. 2006; Tsapakis et al. 2014). By regulating gene expression, drugs may be responsible for brain neuroplasticity, cell survival, or cognitive function (Tardito et al. 2006). Due to this, the search for new methods of pharmacotherapy, also based on genetic research, is becoming more reasonable. Therefore, the purpose of our study was also the evaluation of expression of the selected genes responsible for neuronal transduction and excitability changes in neuronal cells. *Slc6a15* belongs to solute carrier family 6, also referred to as the neurotransmitter-sodium symporter family or Na^+^/Cl^−^-dependent transporters, one of the largest transporter families, which also comprises transporters for monoamine neurotransmitters and gamma-amino butyric acid (GABA) (Kristensen et al. 2011). It is expressed predominantly in neurons with high expression in the cerebral cortex (Cx), hippocampus, and amygdala (Farmer et al. 2000), i.e., brain regions which relevance to depression has been established (Palazidou 2012). *Slc6a15* recognizes neutral amino acids such as proline (Broer et al. 2006), which can be further used in the biosynthesis of glutamate, a neurotransmitter implicated in depression pathophysiology and treatment (Pilc et al. 2013), and has recently emerged as a susceptibility gene for major depression (Kohli et al. 2011). *Comt* encodes intracellularly located enzyme catechol-*O*-methyltransferase (Ulmanen et al. 1997), which degrades catecholamines (Eisenhofer et al. 2004). In the frontal cortex, *Comt* accounts for ca. 60% of the metabolic degradation of dopamine (Karoum et al. 1994), a neurotransmitter which plays a role in antidepressant action (Porcelli et al. 2011). Moreover, *Comt* polymorphisms have been associated with depression (Koike et al. 2018; Wang et al. 2016) and response to antidepressants (Antypa et al. 2013; O’Leary et al. 2014). *Adora1* encodes A_1_ receptor, whose antagonism mediates caffeine-induced wakefulness (Fredholm et al. 2016). A_1_ receptors participate in mediating antidepressant-like effect (Cunha et al. 2015; Serchov et al. 2015). Moreover, sleep deprivation, which represents one of the therapeutic strategies in depression, was shown to be accompanied by an A_1_ receptor upregulation in the human brain (Elmenhorst et al. 2017). In addition to *Comt*, *Slc6a15*, and *Adora1*-relative gene expression, the levels of the studied antidepressants and caffeine in the mice serum and brain were measured.

## Materials and methods

### Animals

The experiment was carried out on 406 naïve adult male Swiss albino mice (25–30 g) purchased from the licensed breeder (Kołacz, Warsaw, Poland). The animals were housed in the environmentally controlled rooms with a 12-h light/dark cycle, in groups of 10 in standard cages under strictly controlled laboratory conditions: temperature maintained at 22–23 °C with relative humidity in a range of 45–55%. Throughout the study, the animals were given ad libitum access to water and food. The experiment began after at least a 1-week acclimation period to the laboratory conditions and was conducted between 8 a.m. and 3 p.m. to minimize circadian influences. Separate groups of animals were used for the forced swim test (FST), tail suspension test (TST), locomotor activity test, and pharmacokinetic studies. Because the locomotor activity test is noninvasive, following its assessment, the tissues were collected for quantitative real-time PCR (qRT-PCR) analysis. All procedures were approved by the Local Ethics Committee at the Medical University of Lublin (license no. 29/2015) and were performed in accordance with binding European standards related to the experimental studies on animal models. Each mouse was used only once.

### Drug administration

Caffeine (5 mg/kg; Sigma-Aldrich, Poznań, Poland) and mianserin hydrochloride (10 mg/kg; Sigma-Aldrich) were dissolved in saline (0.9% sodium chloride (NaCl)). Agomelatine (20 mg/kg; Sigma-Aldrich) was suspended in a 1% aqueous solution of Tween 80 (POCH, Gliwice, Poland). The doses and treatment schedules were selected based on our previous experiments (Poleszak et al. 2016). The mice were randomly assigned to groups that received the solution of caffeine or saline intraperitoneally (i.p.), twice a day (at 8 a.m. and 8 p.m.), for 14 days. Both groups were further divided into subgroups that received on the 15th day caffeine or saline 40 min before behavioral testing and the solutions/suspensions of antidepressants or saline 60 min before behavioral testing. All solutions/suspensions were prepared fresh before administration and were administered at a volume of 0.1 ml per 10 g of body weight. The number of animals used per group was 10 in the FST, TST, and pharmacokinetic studies and 8 in the locomotor activity test.

### Forced swim test

The FST was performed 40 min following caffeine administration and 60 min following drug administration. The procedure was carried out according to the method of Porsolt et al. (1977). Each mouse was placed individually into the glass cylinders (height 25 cm, diameter 10 cm) containing 10 cm of water at 23–25 °C. The animals were left in the cylinder for 6 min. The total duration of immobility was recorded during the last 4 min of the 6-min long testing period. The mouse was judged immobile when it ceased struggling and remained floating motionless in the water, making only the movements necessary to keep its head above the water level. The immobility time was scored in real time by two blind observers. The results are shown as the arithmetic mean of immobility time of animals given in seconds ± standard error of the mean (SEM) for each experimental group.

### Tail suspension test

The TST was performed 40 min following caffeine administration and 60 min following drug administration. The procedure was carried out according to the method of Steru et al. (1985). Each mouse was individually suspended by its tail to a vertical bar in wooden box (30 × 30 cm). The animals were fastened by means of an adhesive tape fixed 2 cm from the end of the tails for 6 min. The total duration of immobility was recorded during the last 4 min of the 6-min long testing period. The mouse was judged immobile when it ceased moving its limbs and body, making only the movements necessary to breathe. The results are shown as the arithmetic mean of immobility time of animals given in seconds ± SEM for each experimental group.

### Spontaneous locomotor activity

The spontaneous locomotor activity was assessed 40 min following caffeine administration and 60 min following drug administration. The spontaneous locomotor activity was measured in an animal activity meter Opto-Varimex-4 Auto-Track (Columbus Instruments, USA). This device consists of four transparent cages with a lid (43 × 43 × 32 cm), a set of four infrared emitters (each emitter has 16 laser beams), and four detectors monitoring animal movements. Mice were placed individually into the cages for 10 min. Spontaneous locomotor activity was evaluated between the 2nd and the 6th min, which corresponds to the time interval analyzed in the FST and the TST. The results are presented as the arithmetic average distance traveled by a mouse in centimeters ± SEM for each experimental group.

### Tissue processing for pharmacokinetic studies

Forty minutes following caffeine administration and 60 min following drug administration, mice were decapitated to collect biological material for pharmacokinetic studies. The blood was collected into Eppendorf tubes and allowed to clot at room temperature. Subsequently, the blood was centrifuged at 5000×*g* for 10 min and serum was collected into polyethylene tubes and frozen at − 25 °C. Immediately after the decapitation, the brains were dissected from the skull, washed with saline, and also frozen at − 25 °C. Serum and brain concentrations of caffeine and the tested antidepressants were assayed by the high-performance liquid chromatography (HPLC) method.

### Determination of antidepressants in serum and brain tissue

The brains were homogenized in distilled water (1:4, *w*/*v*) with a TH220 tissue homogenizer (Omni International, Inc., Warrenton, VA, USA). For agomelatine, 1 ml of brain homogenate or 200 μl of serum was spiked with carbamazepine (100 ng/ml) as an internal standard (IS). Before the extraction, 1 ml of the concentrated NaCl solution (10 g/50 ml) was added to brain homogenate and the samples were vortexed for 15 s. The extraction of agomelatine from brain homogenate was performed using 5 ml of a mixture of dichloromethane/hexane/isoamyl alcohol (39.5:59.5:1 *v*/*v*/*v*), whereas that from serum with 1 ml of dichloromethane. The samples were shaken for 20 min and centrifuged for 15 min at 1000×*g*. After the centrifugation, the organic layers were transferred into conical glass tubes and evaporated to dryness at 37 °C under a gentle stream of nitrogen in a water bath. The residues were dissolved with 100 μl of methanol, and aliquots of 50 μl were injected into the HPLC system. For mianserin, the extraction from serum and brain homogenates was performed using a mixture of ethyl acetate/hexane (30:70, *v*/*v*). Amitriptyline (1 μg/ml) used as IS was added to serum (200 μl) and brain homogenate (0.5 ml) containing mianserin, and the samples were alkalized with 100 and 250 μl of 4 M NaOH, respectively. Next, the samples were extracted with 5 ml of the extraction reagent by shaking for 20 min (IKA Vibrax VXR, Germany). After centrifugation at 1000×*g* for 20 min (Universal 32, Hettich, Germany), the organic layers were transferred to new tubes containing a 150 μl solution of 0.1 M H_2_SO_4_ and methanol (90:10, *v*/*v*), shaken for 0.5 h, and then centrifuged for 15 min (1000×*g*). Then, the organic layers were discarded and 50-μl aliquots of acidic solutions were injected into the HPLC system.

The HPLC system consisted of an isocratic pump (model L-7100) and an autosampler (model L-7200), both from Merck Hitachi (Darmstadt, Germany), and a UV variable-wavelength K-2600 detector (Knauer, Berlin, Germany). Data acquisition and processing were carried out using the D-7000 HSM software (Merck Hitachi). Analysis of agomelatine was performed on a 250 × 4 mm LiChrospher1100 RP-18 column with a particle size of 5 mm (Merck, Darmstadt, Germany) protected with a guard column (4 × 4 mm) with the same packing material, whereas mianserin was determined using a 250 × 4.6 mm Supelcosil LC-CN column with a particle size of 5 μm (Sigma-Aldrich, Steinheim, Germany) protected with a guard column (20 × 4 mm) with the same packing material. The mobile phase consisting of acetonitrile and 50 mM potassium dihydrogen phosphate was mixed at a ratio of 37:63 (*v*/*v*) for agomelatine and 25:75 (*v*/*v*) for mianserin and run at 1 ml/min. Chromatographic analysis was carried out at 21 °C and an analytical wavelength of 230 nm for agomelatine and 214 nm for mianserin.

The calibration curves constructed by plotting the ratio of the peak heights of the studied drug to IS vs. the concentration of the drug were linear in the tested concentration ranges. No interfering peaks were observed in the chromatograms. The assays were reproducible with low intra- and inter-day variation (coefficients of variation less than 10%). The extraction efficiencies of the analyzed compounds and IS ranged from 70 to 92%. Antidepressant concentrations were expressed in nanograms/milliliter of serum or nanograms/gram of wet brain tissue.

### Determination of caffeine in serum and brain tissue

The brains were homogenized in phosphate buffer (pH 7.2; 1:2, *w*/*v*) with a tissue homogenizer (Ultra Turrax T8, IKA, Germany). Caffeine extraction from serum (200 μl) and brain homogenate (300 μl) was performed by 6% perchloric acid protein precipitation. The mixture was vortexed for 30 s and centrifuged at 2500×*g* for 15 min. The supernatants were filtered through a cellulose filter (nominal pore diameter 0.20 μm), and a 20 μl volume of each sample solution was injected into the HPLC system. The undertaken sample preparation method was the modification of the procedure described by Novitskayaa et al. (2013).

The HPLC system (PerkinElmer Series 200, Shelton, CT, USA) consisted of an isocratic pump, a variable-wavelength UV-Vis detector, and an autosampler. All analyses were performed on a 250 × 4.6 mm Hypersil^®^ column with a particle size of 5 μm (Thermo Electron Corporation, Waltham, MA, USA) protected with a guard column (4 × 4 mm) with the same packing material. The mobile phase consisting of water (brought to pH 4.0 with 1% formic acid)/acetonitrile/methanol (80:8:14, *v*/*v*/*v*) was run at 1 ml/min. Chromatographic analysis was carried out at 21 °C and an analytical wavelength of 273 nm.

The calibration curves constructed based on the analysis of samples containing caffeine at concentrations covering the range of 1 to 24 μg/ml prepared for murine serum and brain homogenates were linear in the tested concentration ranges. No interference from the matrix at the retention time of caffeine was observed in the chromatograms. Caffeine concentrations were expressed in micrograms/milliliter of serum or nanograms/gram of wet brain tissue.

### The quantitative real-time PCR analysis

Because locomotor activity is a noninvasive test, following its assessment, mice were decapitated; their brains were rapidly dissected and immersed in cooled (2–8 °C) saline. The Cx was dissected on a cold plate, immediately frozen on dry ice, and stored at − 80 °C until analysis.

The qRT-PCR method was used to evaluate the expression of the selected genes in the Cx. RNA was isolated from 30 mg of tissue using Syngen Tissue RNA Mini Kit (Syngen Biotech, Poland), and reverse transcription was performed by means of NG dART RT-PCR kit (EURx, Poland) according to the manufacturer’s instructions. The relative expression of the following genes: *Slc6a15*, *Comt*, and *Adora1* (Mn00558415_m1, Mn00514377_m1, Mn01308023_m1, respectively (TaqMan Gene Expression Assays, Life Technologies, USA)) was determined by qRT-PCR and the ΔΔCt method using hypoxanthine-guanine phosphoribosyltransferase (HGPRT) (Mn00446968_m1) as an endogenous control. The reaction was carried out in quadruplicate using the SmartChip Real-Time PCR System (WaferGen Biosystems) and TaqMan Fast Universal PCR Master Mix (2×) (Applied Biosystems, USA) according to the manufacturer’s instructions. The data quality screening based on amplification curves and Ct values was performed to remove any outlier data before ΔΔCt calculations and to determine fold change in messenger RNA (mRNA) levels. Statistical analysis was performed on RQ values (RQ = 2 − ΔΔCt).

### Statistical analysis

The results obtained in the FST, TST, and locomotor activity tests and relative gene expression levels of *Slc6a15*, *Comt*, and *Adora1* were analyzed using a two-way ANOVA with chronic treatment (saline chronic vs. caffeine chronic) and 15th-day treatment as factors, followed by a Tukey’s post hoc test (for the comparison of effects within chronic treatment) or a Sidak’s post hoc test (for the comparison of effects within 15th-day treatments). The concentrations of caffeine and the tested antidepressants in serum and brains of mice were analyzed using a Student’s *t* test. The statistical analysis was carried out using GraphPad Prism for Windows, version 7.04 (GraphPad Software, San Diego, CA, USA). All results are presented as the mean ± SEM. A *p* value < 0.05 was considered as statistically significant with 95% confidence.

## Results

### Forced swim test and tail suspension test

**Fig. 1:**
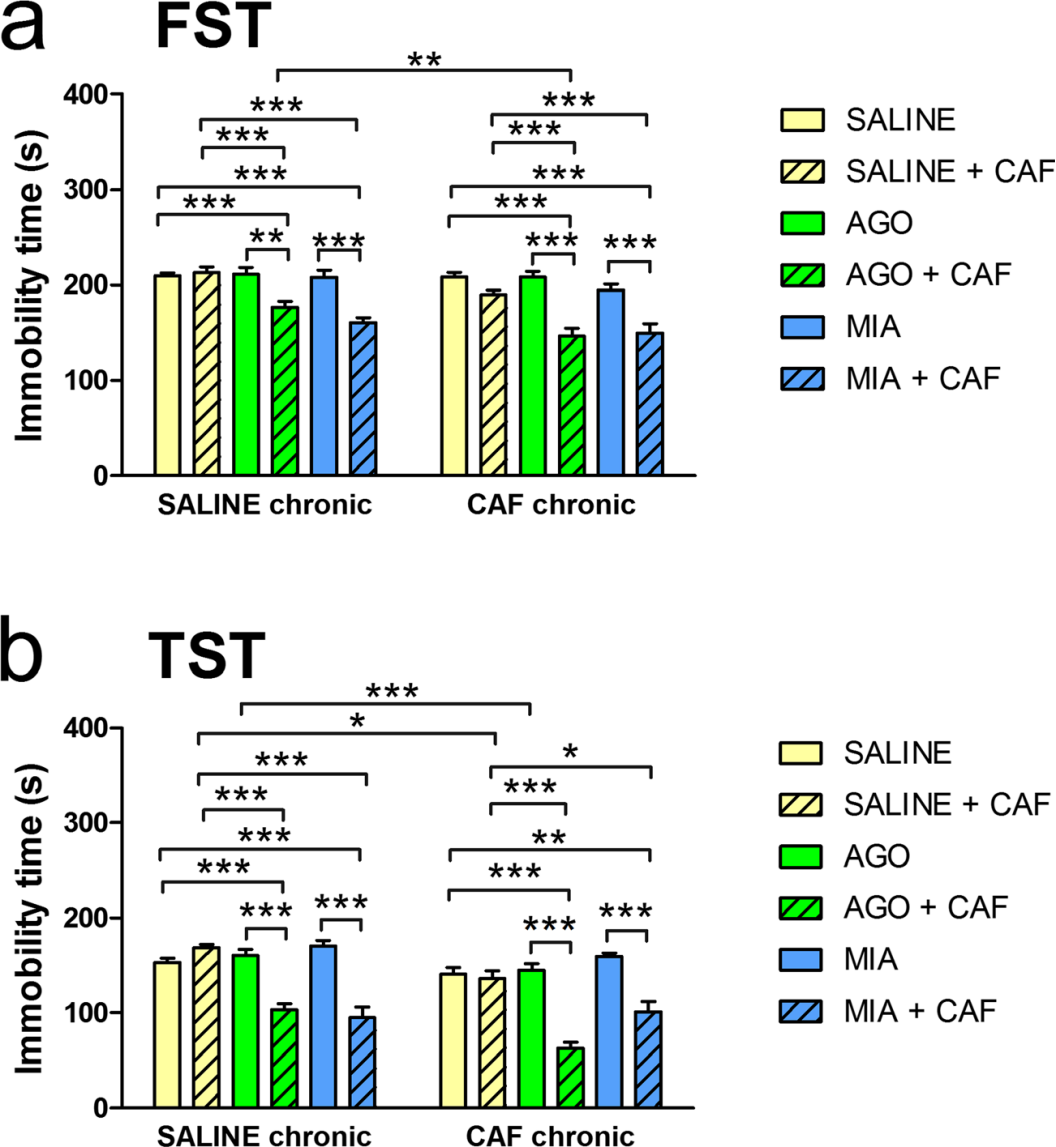
The effects of chronic (14-day) caffeine (CAF) (5 mg/kg, twice daily, i.p.) administration and 15th-day treatment: antidepressant drug agomelatine (AGO) (20 mg/kg, i.p.) or mianserin (MIA) (10 mg/kg, i.p.) alone or simultaneously with caffeine (5 mg/kg, i.p.) in the forced swim test (FST) (**a**) and the tail suspension test (TST) (**b**) in mice. *p < 0.05, **p < 0.01, ***p < 0.001, by the two-way ANOVA followed by a Tukey’s or Sidak’s post hoc test; values expressed as the mean + SEM

The effects of chronic (14-day) caffeine administration and 15th-day treatment (antidepressant drug treatment alone or simultaneously with caffeine) on the total duration of immobility in the FST are shown in Fig. 1a, and those in the TST are shown in Fig. 1b. In the FST, a two-way ANOVA showed a significant effect of chronic treatment [*F*(1,117) = 14.8, *p* = 0.0002], a significant effect of 15th-day treatment [*F*(5,117) = 32.64, *p* < 0.0001], and a not significant chronic × 15th-day treatment interaction [*F*(5,117) = 1.76, *p* = 0.1266]. In the TST, a two-way ANOVA showed a significant effect of chronic treatment [*F*(1,99) = 18.17, *p* < 0.0001], a significant effect of 15th-day treatment [*F*(5,99) = 45.13, *p* < 0.0001], and a significant chronic × 15th-day treatment interaction [*F*(5,99) = 2.696, *p* = 0.0251]. In mice chronically treated with saline, during the FST as well as during the TST, administration of caffeine simultaneously with agomelatine or mianserin significantly decreased the total duration of immobility as compared to mice that received saline of the 15th day or mice that received caffeine on the 15th day or mice that received the respective antidepressant on the 15th day. Also in mice chronically treated with caffeine, during the FST as well as during the TST, administration of caffeine simultaneously with agomelatine or mianserin significantly decreased the total duration of immobility as compared to mice that received saline on the 15th day or mice that received caffeine on the 15th day or mice that received the respective antidepressant on the 15th day. In the FST and TST, there were no changes in the immobility time between mice that received only an antidepressant drug on the 15th day and mice that received saline on the 15th day, in mice chronically treated with saline as well as in mice chronically treated with caffeine. There were no significant changes in the immobility time between mice chronically treated with saline and mice chronically treated with caffeine in the FST and TST. The immobility time, however, was shorter in mice that received agomelatine and caffeine following chronic administration of caffeine than in mice that received the above combination following chronic administration of saline. Additionally, in the TST, the immobility time was shorter in mice that received caffeine on day 15 following chronic administration of caffeine than in mice that received caffeine on day 15 following chronic administration of saline.

### Spontaneous locomotor activity

The effects of chronic caffeine administration and antidepressant drug treatment alone or simultaneously with caffeine on the spontaneous locomotor activity of mice are shown in Table 1. A two-way ANOVA demonstrated a significant effect of 15th-day treatment [*F*(5,83) = 8.706, *p* < 0.0001], a not significant effect of chronic treatment [*F*(1,83) = 1.571, *p* = 0.2135], and a not significant chronic × 15th-day treatment interaction [*F*(5,83) = 0.3936, *p* = 0.8519]. Administration of mianserin significantly decreased the spontaneous locomotor activity compared to saline in mice chronically treated with saline. There were no significant changes in the spontaneous locomotor activity between other treatment groups.

**Table 1:** The effects of chronic (14-day) caffeine (CAF) (5 mg/kg, twice daily, i.p.) administration and 15th-day treatment: antidepressant drug agomelatine (AGO) (20 mg/kg, i.p.) or mianserin (MIA) (10 mg/kg, i.p.) alone or simultaneously with caffeine (5 mg/kg, i.p.) on the spontaneous locomotor activity of mice

Treatment (mg/kg)		Distance traveled (cm)
Twice daily for 14 days	15th day	
Saline	Saline	578.0 ± 47.66
Saline	Saline + CAF 5	647.1 ± 46.17
Saline	AGO 20	459.3 ± 55.16
Saline	AGO 20 + CAF 5	627.3 ± 60.10
Saline	MIA 10	275.6 ± 39.47**
Saline	MIA 10 + CAF 5	375.3 ± 71.4
CAF 5	Saline	561.4 ± 50.53
CAF 5	Saline + CAF 5	649.8 ± 91.02
CAF 5	AGO 20	551.5 ± 53.2
CAF 5	AGO 20 + CAF 5	681.0 ± 39.62
CAF 5	MIA 10	284.5 ± 83.89
CAF 5	MIA 10 + CAF 5	520.6 ± 124.4

Distance traveled was recorded between the 2nd and the 6th min of the test. Each experimental group consisted of six to eight animals

**p < 0.01, vs. the saline + saline group (two-way ANOVA followed by a Tukey’s post hoc test; values expressed as the mean ± SEM)

### Pharmacokinetic studies

The effects of caffeine and drug treatment on serum and brain concentrations of the tested antidepressants given on the 15th day after a 14-day administration of caffeine are shown in Table 2. In the case of co-administration of caffeine and mianserin on the 15th day after chronic administration of caffeine, a decreased mianserin concentration in serum (*t* test: *p* = 0.0001) was observed in comparison to the mice treated with mianserin alone. The concentration of this drug in the brain tissue was higher, but the difference was not significant (*t* test: *p* = 0.8833). Similarly, increased agomelatine concentrations in both serum and brain tissue were observed in the group treated with caffeine and agomelatine on the 15th day after chronic administration of caffeine vs. the group receiving only the antidepressant drug on this day, but the changes were not significant (*t* test: *p* = 0.3660 and *p* = 0.3149, respectively).

**Table 2:** The effects of chronic caffeine administration on mouse serum and brain concentrations of antidepressant drugs (agomelatine, mianserin) given alone or simultaneously with caffeine

Treatment (mg/kg)		Drug concentration	
Twice daily for 14 days	15th day	Serum (ng/ml)	Brain (ng/g)
Caffeine 5	Agomelatine 20	267.0 ± 70.82	288.3 ± 78.77
Caffeine 5	Caffeine 5 + agomelatine 20	432.2 ± 162.8	526.5 ± 215.7
Caffeine 5	Mianserin 10	370.2 ± 33.43	6671 ± 806.8
Caffeine 5	Caffeine 5 + mianserin 10	153.8 ± 23.55***	6879 ± 1165

Each experimental group consisted of 8–10 animals. Results are presented as mean values ± SEM

***p < 0.001, compared with the respective control group (Student’s *t* test)

The effects of tested drugs on serum and brain concentrations of caffeine given on the 15th day after chronic administration of caffeine in mice are shown in Table 3. In the case of joint administration of caffeine and mianserin on the 15th day after chronic administration of caffeine, a significant increase in caffeine concentration in serum (*t* test: *p* = 0.0031) but not in the brain tissue (*t* test: *p* = 0.2317) was observed. Co-administration of caffeine and agomelatine on the 15th day after chronic administration of caffeine resulted in an increase in caffeine concentration in brain tissue (*t* test: *p* = 0.0025) and a nonsignificant increase in murine serum (*t* test: *p* = 0.0707).

**Table 3:** The effects of chronic caffeine administration and antidepressant drug (agomelatine, mianserin) treatment simultaneously with caffeine on the concentrations of caffeine in mouse serum and brain

Treatment (mg/kg)		Caffeine concentration	
Twice daily for 14 days	15th day	Serum (­μg/ml)	Brain (ng/g)
Caffeine 5	Caffeine 5 + saline	3.030 ± 0.3710	1079 ± 180.9
Caffeine 5	Caffeine 5 + agomelatine 20	3.706 ± 0.1589	2447 ± 326.1**
Caffeine 5	Caffeine 5 + mianserin 10	4.229 ± 0.1479**	861.8 ± 61.58

Each experimental group consisted of 8–10 animals. Results are presented as mean values ± SEM

**p < 0.01, compared with the respective control group (Student’s *t* test)

### Gene expression studies

**Fig. 2:**
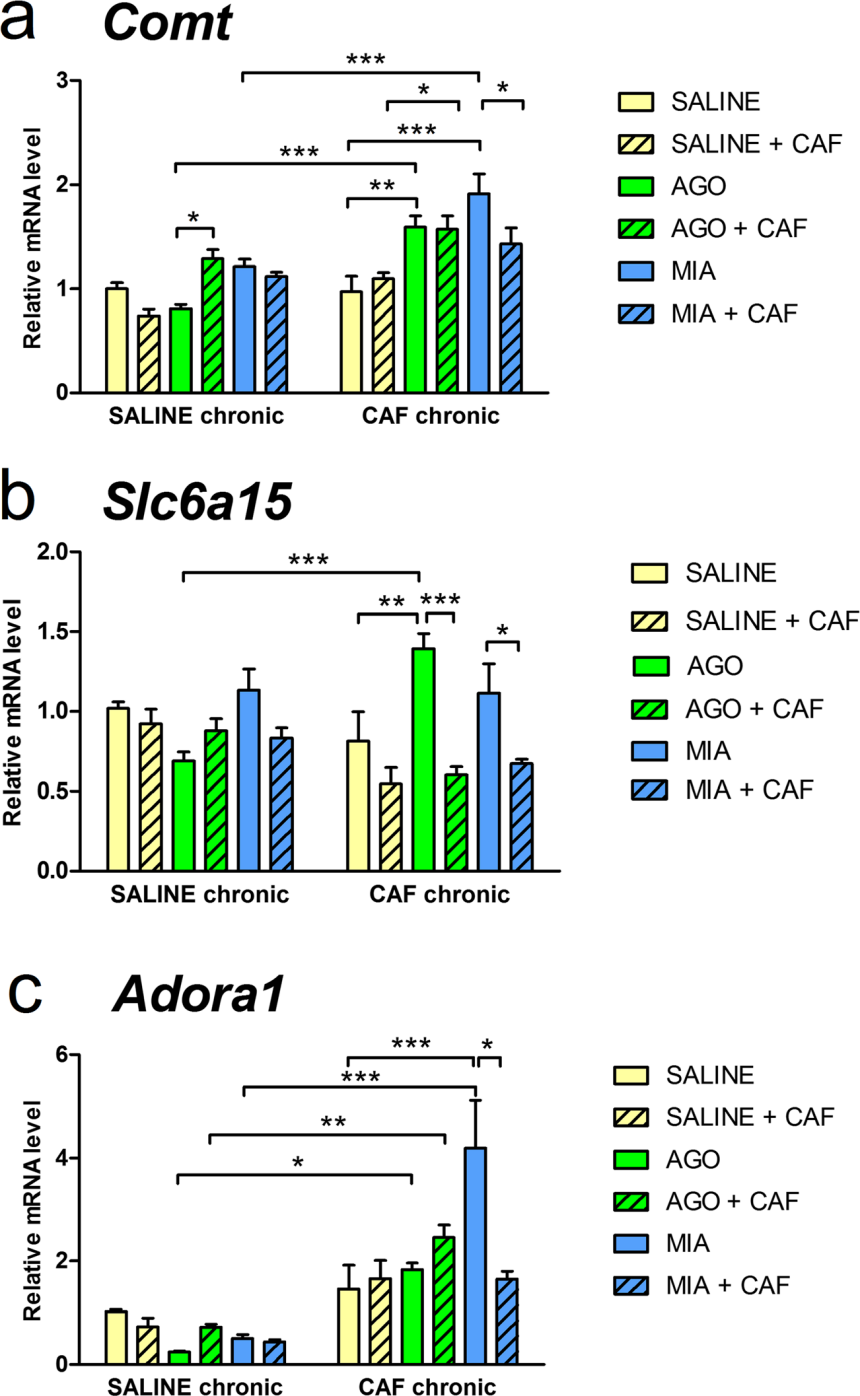
The effects of chronic (14-day) caffeine (CAF) (5 mg/kg, twice daily, i.p.) administration and 15th-day treatment: antidepressant drug agomelatine (AGO) (20 mg/kg, i.p.) or mianserin (MIA) (10 mg/kg, i.p.) alone or simultaneously with caffeine (5 mg/kg, i.p.) on the mRNA level of catechol-*O*-methyltransferase (*Comt*) (**a**); solute carrier family 6, member 15 (*Slc6a15*) (**b**); and adenosine A1 receptor (*Adora1*) (**c**) in the cerebral cortex (Cx). *p < 0.05, **p < 0.01, ***p < 0.001, by the two-way ANOVA followed by a Tukey’s or Sidak’s post hoc test; values expressed as the mean + SEM

The effects of chronic (14-day) caffeine administration and 15th-day treatment (antidepressant drug treatment alone or simultaneously with caffeine) on the mRNA level of *Comt* in the Cx are shown in Fig. 2a. A two-way ANOVA showed a significant effect of chronic treatment [*F*(1,51) = 43.17, *p* < 0.0001], a significant effect of 15th-day treatment [*F*(5,51) = 11.25, *p* < 0.0001], and a significant chronic × 15th-day treatment interaction [*F*(5,51) = 4.018, *p* = 0.0038]. There were no significant differences in *Comt* mRNA level in the Cx between mice that received saline and mice that received caffeine for 14 days, but *Comt* mRNA level was higher in mice that received agomelatine or mianserin following chronic treatment with caffeine, compared to mice that received the respective antidepressants following treatment with saline. In mice chronically treated with saline, administration of agomelatine simultaneously with caffeine significantly increased *Comt* mRNA level in the Cx, compared to mice that received agomelatine without caffeine on the 15th day. In mice chronically treated with caffeine, administration of agomelatine or mianserin on day 15 significantly increased *Comt* mRNA level compared to the group that received saline on this day. Moreover, in mice chronically treated with caffeine, co-administration of agomelatine and caffeine on day 15 induced significantly increased *Comt* mRNA level compared to mice that received only caffeine on this day. Furthermore, in mice chronically treated with caffeine, co-administration of caffeine and mianserin on day 15 significantly decreased *Comt* mRNA level compared to mice that received only mianserin on this day.

The effects of chronic caffeine administration and antidepressant drug treatment alone or simultaneously with caffeine on the mRNA level of *Slc6a15* in the Cx are shown in Fig. 2b. A two-way ANOVA showed a significant effect of 15th-day treatment [*F*(5,52) = 5.372, *p* = 0.0005] and a significant chronic × 15th-day treatment interaction [*F*(5,52) = 7.204, *p* < 0.0001], but no effect of chronic treatment [*F*(1,52) = 0.885, *p* = 0.3512]. There were no significant differences in *Slc6a15* mRNA level in the Cx between mice that received saline and mice that received caffeine for 14 days, but *Slc6a15* mRNA level was higher in mice that received agomelatine following chronic treatment with caffeine, compared to mice that received this drug following treatment with saline. In mice chronically treated with saline, no significant changes in *Slc6a15* mRNA level between the 15th-day treatment subgroups were observed. In mice chronically treated with caffeine, administration of agomelatine on the 15th day significantly increased *Slc6a15* mRNA level compared to mice that received saline on this day. Moreover, in mice that received caffeine for 14 days, co-administration of caffeine and agomelatine or mianserin on day 15 significantly decreased *Slc6a15* mRNA level, compared to mice that received only the respective antidepressant on this day.

The effects of chronic caffeine administration and antidepressant drug treatment alone or simultaneously with caffeine on the mRNA level of *Adora1* in the Cx are shown in Fig. 2c. A two-way ANOVA showed a significant effect of chronic treatment [*F*(1,52) = 75.49, *p* < 0.0001], a significant effect of 15th-day treatment [*F*(5,52) = 4.972, *p* = 0.0009], and a significant chronic × 15th-day treatment interaction [*F*(5,52) = 6.268, *p* = 0.0001]. There were no significant differences in *Adora1* mRNA level in the Cx between mice that received saline for 14 days and mice that received caffeine for 14 days, but *Adora1* mRNA level was higher in mice that received agomelatine, agomelatine and caffeine, or mianserin following chronic treatment with caffeine, compared to mice that received agomelatine, agomelatine and caffeine, or mianserin, respectively, following treatment with saline. In mice chronically treated with caffeine, administration of mianserin on the 15th day induced significantly increased *Adora1* mRNA level compared to mice that received saline on this day. Moreover, in mice chronically treated with caffeine, co-administration of caffeine and mianserin on day 15 significantly decreased *Adora1* mRNA level, compared to mice that received only mianserin on this day.

## Discussion

Outpatients with major depression are more likely to report increased caffeine intake in response to depressive symptoms than healthy volunteers (Leibenluft et al. 1993). Moreover, it was shown that 13% of psychiatric patients present a high (≥ 750 mg daily) caffeine consumption, although the intake of caffeine in patients with major depression was lower than in schizophrenic patients (Rihs et al. 1996). Noteworthy, a prospective cohort study with a follow-up period of 17 years, which included middle-aged, depressive-free at-baseline men, demonstrated a significant inverse correlation between coffee drinking and the development of depression. However, no such correlation was shown with tea or caffeine consumption (Ruusunen et al. 2010). Also, a large prospective cohort of older women, free from depression at baseline, demonstrated a decreased risk of developing depression with increasing consumption of caffeinated coffee (Lucas et al. 2011). These data show a link between coffee/caffeine intake and depression occurrence/development.

We have previously shown that acute administration of caffeine at doses of 10, 20, and 50 mg/kg produced an antidepressant-like effect in the FST in mice and did not affect the spontaneous locomotor activity (Szopa et al. 2016). Moreover, caffeine at a dose of 5 mg/kg did not induce an antidepressant-like effect in this test but potentiated the activity of antidepressant drugs belonging to almost all known antidepressant classes (i.e., imipramine, desipramine, fluoxetine, paroxetine, escitalopram, reboxetine, venlafaxine, mianserin, milnacipran, bupropion, agomelatine, and moclobemide) (Kale and Addepalli 2014; Poleszak et al. 2016; Poleszak et al. 2015; Szopa et al. 2016). This observation raises the possibility of augmentation of antidepressant therapy with caffeine. Of note, recent clinical study indicated beneficial effects of caffeine in terms of augmenting antidepressant treatment. A 2-week supplementation with a low dose of caffeine (60 mg/day) enhanced the efficacy of treatment with a selective serotonin reuptake inhibitor (SSRI), escitalopram (Liu et al. 2017). Since in the present study no increases in the spontaneous locomotor activity were found, the results indicate an antidepressant-like activity of low, ineffective doses of agomelatine or mianserin administered together with caffeine. Thus, our data further support the possibility of augmenting of antidepressant therapy with caffeine. However, the present results suggest that chronic treatment with caffeine followed by its withdrawal may not be sufficient to increase the effects of antidepressant drugs, in contrast to concomitant administration of caffeine and antidepressants following chronic exposure to caffeine. It should be stressed that the majority of large studies failed to demonstrate major health hazards of coffee or tea consumption (Fredholm et al. 2016), while it has been well characterized that caffeine withdrawal may produce depressed mood (Dews et al. 2002; Juliano and Griffiths 2004; Nehlig et al. 1992).

We have previously shown that acute administration of caffeine at a dose of 5 mg/kg did not result in significant changes in the concentrations of agomelatine or mianserin in serum or brain tissue, but a significant decrease in the level of caffeine following joint administration with mianserin was observed (Poleszak et al. 2016). Here, we found that caffeine administered chronically slightly increased the concentration of agomelatine in the biological material. Moreover, in this drug combination, a considerable increase in the level of caffeine in the brain tissue, but only small changes in serum caffeine concentration, was observed. It is possible that the observed alterations are associated with the modifications in biotransformation of caffeine, since both caffeine and agomelatine are metabolized mainly via hepatic CYP1A2 (Begas et al. 2007). These changes suggest that a caffeine-agomelatine interaction presumably is pharmacokinetic in nature. The interplay between caffeine and mianserin demonstrated in the course of the present study is not entirely clear. A significant increase in the concentration of caffeine and a decrease in the concentration of mianserin in serum were observed after concomitant administration of these two agents. Surprisingly, changes in mianserin and caffeine levels in serum were not parallel to alterations in their concentrations in brain tissue. The concentration changes observed in the biological materials after concurrent administration of atypical antidepressant drugs and caffeine may be associated with dose-dependent metabolism of caffeine (Denaro et al. 1990), and the use of a low dose of caffeine may result in drug interactions that were not very explicit (Kale and Addepalli 2014). The results obtained suggest that in this drug combination, the caffeine-mianserin interaction might have been partially pharmacodynamic and pharmacokinetic in nature.

Moreover, the antidepressant-like effect observed after joint administration of agomelatine or mianserin and caffeine on day 15 in mice chronically treated with caffeine was associated with a decreased expression of *Slc6a15* at mRNA level in the Cx. We have previously demonstrated that co-administration of single doses of SSRIs, fluoxetine or escitalopram, and caffeine in mice chronically (for 2 weeks) treated with caffeine led to a decreased *Slc6a15* level in the Cx, which was associated with an antidepressant-like effect in the FST and TST (Szopa et al. 2017). Another group has shown that chronic (14-day) treatment with low (3 mg/kg) or high (10 mg/kg) dose of fluoxetine caused an upregulation of *Slc6a15* at mRNA level in the hippocampus (Hagglund et al. 2013), which points to the role of this gene in the mechanism of action of this drug. While our previous data suggested the involvement of *Slc6a15* in the mechanism of action of SSRIs given together with caffeine following a chronic period of caffeine administration, the current data add a new evidence to the role of this gene in the mode of action of caffeine and drugs belonging to other classes of antidepressants.

Of note, *Slc6a15* knockout animals displayed lower levels of depressive-like behavior following chronic stress, while *Slc6a15* overexpression animals displayed an increase in depressive-like behavior (Santarelli et al. 2016). These data show that a reduction of *Slc6a15* expression may provide protection against the detrimental effects of chronic stress on behavior. Moreover, expression of *Slc6a15* was shown to correlate with proline and glutamate content of the hippocampus. Furthermore, *Slc6a15* overexpression animals displayed an increased GluR1 subunit of the glutamate α-amino-3-hydroxy-5-methyl-4-isoxazolepropionic acid (AMPA) receptor mRNA expression in subregions of the hippocampus (Santarelli et al. 2016). Increasing evidence suggests that conventional antidepressants (Reus et al. 2016) and newer antidepressants like agomelatine (Tardito et al. 2012) exert effects on the glutamatergic system and this action may be related to their therapeutic activity. Agomelatine or fluoxetine was found to abolish the depolarization-evoked increase in glutamate release induced by acute foot shock stress from synaptosomes of the prefrontal/frontal cortex (Musazzi et al. 2010). Thus, *Slc6a15* may be a link between antidepressants with different mechanisms of action (SSRIs, agomelatine, mianserin) and the glutamatergic system with regard to antidepressant-like effect.

Administration of ineffective doses of caffeine (which acts as a nonselective A_1_ and A_2A_ receptor antagonist) enhanced the activity of ineffective doses of the following antidepressants: imipramine, desipramine, fluoxetine, paroxetine, escitalopram, reboxetine, venlafaxine, mianserin, milnacipran, bupropion, agomelatine, and moclobemide in the FST (Kale and Addepalli 2014; Poleszak et al. 2016; Poleszak et al. 2015; Szopa et al. 2016). On the other hand, the antidepressant-like effect of creatine and ketamine, fast-acting antidepressant agents targeting the glutamatergic system, but not fluoxetine, was abolished by caffeine, DPCPX (a selective adenosine A_1_ receptor antagonist), and ZM241385 (a selective adenosine A_2A_ receptor antagonist), while the administration of ineffective doses of creatine or ketamine combined with CHA (a selective adenosine A_1_ receptor agonist), DPMA (a selective adenosine A_2A_ receptor agonist), or dipyridamole (an adenosine transporter inhibitor) produced a synergistic antidepressant-like effect in the TST (Cunha et al. 2015). It was also shown that increasing A_1_ receptor expression evokes resilience against a depressive-like behavior in the FST and TST as well as exerts an antidepressant-like effect in a chronic stress model (Serchov et al. 2015). These data suggest that antidepressant-like effect is related to activation of A_1_ receptors, but this observation may not be relevant to classic antidepressants in view of the fact that DPCPX and ZM241385 did not prevent the anti-immobility effect of fluoxetine in the FST (Cunha et al. 2015). Of note, in a chronic stress model of depression, contradictory results on the effectiveness of classic antidepressants and an agent transformed in vivo to endogenous glutamate *N*-methyl-d-aspartate (NMDA) receptor antagonist, kynurenic acid, were shown (Biagini et al. 1993). Given wakefulness-promoting effects of caffeine, a higher level of *Adora1* in mice that received agomelatine, agomelatine and caffeine, or mianserin following chronic treatment with caffeine, compared to mice that received the respective drug or its combination with caffeine following chronic treatment with saline, seems to be convergent with the observation that sleep deprivation is accompanied by an A_1_ receptor upregulation (Elmenhorst et al. 2017). However, here, the antidepressant-like effect observed after concomitant administration of caffeine and mianserin on day 15 after a 14-day treatment with caffeine was parallel to the decreased *Adora1* mRNA level in the Cx. On the other hand, a study by Uzbay et al. (2007) showed that the anticonvulsant effect of tianeptine, an atypical antidepressant, is mediated by the activation of A_1_ receptor. Our data point to the involvement of A_1_ receptors in the mechanism of action of mianserin as well as in the mechanism underlying beneficial effects of caffeine on the antidepressant treatment. We have previously demonstrated that decreased *Adora1* expression in the Cx was parallel to the antidepressant-like effect of joint administration of caffeine and escitalopram, but not fluoxetine, in mice chronically treated with caffeine (Szopa et al. 2017).

In the present study, in mice treated for 14 days with caffeine, administration of mianserin on day 15 without caffeine increased the expression of *Comt* and did not produce an antidepressant-like effect, whereas administration of mianserin together with caffeine on day 15 decreased the mRNA level of *Comt*, compared to mianserin given alone, which was parallel to the antidepressant-like effect. *Comt*, an intracellular enzyme located in the postsynaptic membrane of neurons, participates in the degradation of dopamine, adrenaline, and noradrenaline. Antidepressants by increasing catecholamine metabolism may affect the activity of enzymes involved in their metabolism (Gogos et al. 1998). A higher level of *Comt* may result in a higher activity of the enzyme encoded by this gene and may lead to the decreased levels of catecholamine neurotransmitters and, thus, the lack of antidepressant-like effect, whereas decreased *Comt* level may lead to the opposite effect. Hence, in addition to *Adora1* and *Slc6a15*, *Comt* may be involved in beneficial effects observed after concomitant administration of caffeine and mianserin in animals chronically treated with caffeine. In contrast, in saline-treated rats, antidepressant-like effects observed after joint administration of agomelatine and caffeine were parallel to the increased *Comt* mRNA level in the Cx, which may be due to differences in the primary mechanism of action between agomelatine and mianserin.

## Conclusions

Our findings show an antidepressant-like activity of single, per se ineffective doses of agomelatine or mianserin in mice in which caffeine was given in addition to respective antidepressants following a period of chronic (14-day) treatment with caffeine, in contrast to the mice, in which caffeine was withdrawn after a 14-day period of its chronic administration. Thus, the present study further supports the possibility of augmenting the antidepressant therapy with caffeine. Our data point to the link between antidepressant-like effect and decreased *Slc6a15* expression in the Cx. Based on our current and previous data, we suggest that *Slc6a15* may be involved in the mechanism underlying antidepressant-like effect of joint administration of caffeine and antidepressants independent on the primary mechanism of action of the antidepressant drug. Moreover, the antidepressant-like effect observed after concomitant administration of mianserin and caffeine in mice chronically treated with caffeine may be related to a decreased *Adora1* and *Comt* expression in the Cx. The changes in *Adora1*, *Slc6a15*, and *Comt* expression may result in changes in the function of encoded proteins and subsequent alterations within adenosine, glutamatergic, and monoaminergic systems, all of which are critically involved in depression pathophysiology and treatment.
